# Brain natriuretic peptide measurements using standard biochemical equipment: Comparisons with conventional immunoassays

**DOI:** 10.1371/journal.pone.0268895

**Published:** 2022-05-24

**Authors:** Yukie Higa, Yosuke Nabeshima, Tetsuji Kitano, Masaharu Kataoka, Akemi Nakazono, Masaaki Takeuchi

**Affiliations:** 1 Department of Laboratory and Transfusion Medicine, University of Occupational and Environmental Health Hospital, Kitakyushu, Japan; 2 Second Department of Internal Medicine, University of Occupational and Environmental Health, Kitakyushu, Japan; 3 Department of Cardiology and Nephrology, Wakamatsu Hospital of University of Occupational and Environmental Health, Kitakyushu, Japan; Scuola Superiore Sant’Anna, ITALY

## Abstract

**Background:**

Brain natriuretic peptide (BNP) is an essential cardiac biomarker for diagnosing heart failure and for prognoses in patients with various cardiac diseases. However, measurement requires immunological assays that are not available in every hospital. Recently, a novel BNP kit (Nanopia BNP-A, Sekisui Inc.; BNPn) that uses general-purpose, automated, biochemical analyzers has become commercially available. We assessed how its accuracy and utility compare with those of conventional immunological tests.

**Methods and results:**

We retrospectively collected 1491 conventional BNP measurements (BNPc), which had been clinically indicated for BNP testing and for which residual samples were still stored in the laboratory. We measured BNP using the novel kit and determined the correlation of BNP levels between the two methods. We also assessed the predictive value of both BNP measurements for major cardiac events (MACEs). The analytical performance of both measuring methods was similar. Log-transformed BNP measured by both methods showed strong correlation (r = 0.92); however, log-transformed BNPn was significantly higher than log-transformed BNPc (p<0.001). BNPc of 200 ng/L was used to stratify patients into two groups. According to the regression formula between the two methods, we determined a cut-off value of BNPn as 250 ng/L. During a median of 15 months of follow-up, 43 MACEs developed. Both BNPc and BNPn were associated with MACEs. Kaplan-Meier survival analysis indicated that both BNPc and BNPn cut-off values stratified the high-risk group for prognostication. The diagnostic and prognostic utilities were proven even if the lower cut-off values (BNPc = 100 ng/L, BNPn = 130 ng/L) were employed.

**Conclusions:**

A new BNP measurement using biochemical equipment provides prognostic value similar to that of conventional BNP analysis; thus, it should prove useful in hospitals in which conventional immunological examinations are not available.

## Introduction

Brain natriuretic peptide (BNP) is an established biomarker for diagnosing heart failure (HF) and for predicting outcomes of patients with HF, ischemic heart disease, and valvular heart disease [1–4]. Generally, the plasma BNP concentration is measured using a chemiluminescent enzyme immunoassay kit or a fluorescence immunoassay kit [5–7]. Those methods are established and widely used for rapid BNP testing; however, they require an immunoassay analyzer, which is not available in every hospital, especially small- to medium-sized Japanese hospitals. Therefore, physicians working in such hospitals cannot determine patient plasma BNP levels immediately, making it impossible to use BNP for rapid diagnosis of HF. This situation can adversely affect timely diagnosis and subsequent treatment, resulting in poor outcomes. Recently, a novel BNP kit using general-purpose automatic biochemical analyzers has become commercially available. If the method is comparably accurate and reliable, these measurements may eliminate the aforementioned limitations. Accordingly, the aims of this study were: 1) to compare BNP levels between the conventional and novel methods; 2) to compare the prognostic value of BNP concentrations based on the novel method with those of the standard method.

## Methods

### Study population

This was a retrospective observational study at a single center in Japan. The institutional review board of the School of Medicine of the University of Occupational and Environmental Health approved the study, and the need for informed consent was waived. From January, 2019 to August, 2020, there were 1498 clinically indicated BNP measurements, and 1491 residual plasma samples were still stored at -80°C. Among 1491 samples, 273 plasma samples corresponded to repeated testing; thus, 1218 patients received at least one BNP measurement. Finally, 1204 patients were targets of prognostic analyses because follow-up data could not be obtained for 14 patients (1%).

### BNP measurements

Ethylenediaminetetraacetic acid (EDTA) tubes were used for blood sampling. Specimens collected in EDTA tubes were centrifuged, and the separated plasma was immediately dispensed into another plastic tube. Using this separated plasma, BNP was measured with a commercially available assay and an immunoassay analyzer (Centaur XP, Siemens Inc., Tokyo, Japan). Residual plasma was then frozen and again stored at -80°C. A median of 251 days (interquartile range [IQR]: 207 to 299 days) after storage, frozen plasma samples were allowed to thaw for 5 min under running water, and BNP concentrations were re-determined using a novel kit for general-purpose, automatic biochemical analyzers (TBA-FX8, Canon Medical Systems, Tochigi, Japan), which is based on latex agglutination and turbidimetric absorbance readings (Nanopia BNP-A, Sekisui Medical Co., Ltd., Tokyo, Japan). BNP measurements using the novel method were performed during consecutive 12 working days.

### Follow-up

Follow-up information was obtained from electronic medical records. The primary outcome was a composite of major adverse cardiac events (MACEs), including cardiac death, non-fatal myocardial infarction, HF requiring hospitalization, and ventricular tachyarrhythmia. The total follow-up time was calculated from the first date of blood sampling to the first event or to the maximum length of follow-up for those without MACEs.

### Statistical methods

Continuous data are expressed as means ± SD or medians and IQRs, according to the data distribution. Categorical data are presented as numbers or percentages. BNP levels were log-transformed. Paired comparisons were performed using paired t-tests or Wilcoxon Signed Rank tests. The concordance rate was calculated between groups stratified by BNP level using the conventional method (BNPc = 200 ng/L) and BNP level using the novel method (BNPn = 250 ng/L). Diagnostic accuracy of MACEs predicted by BNPc and BNPn were compared using McNemar’s test. The area under the curve (AUC) of the receiver operating characteristic (ROC) curve was used to assess diagnostic usefulness, and DeLong’s test was conducted to compare AUCs by the two methods. Survival analysis was performed with Kaplan-Meyer survival analysis. Cox proportional hazard regression analysis was performed to calculate hazard ratios and their 95% confidence intervals. To see the availability of early diagnosis of HF, we performed the same analyses with another cut-off (BNPc = 100 ng/L, BNPn = 130 ng/L).

We also evaluated the analytical performance of the two BNP assays with reference to a previous study based on BS ISO 11843–1:1997 and IUPAC guidelines [8]. We used physiological saline as the blank of the method (BoM). We repeatedly measured BoM values in different runs and estimated the distribution of BoM. The limit of blank (LoB) was calculated by the following formula: LoB = mean of BoM + 1.645 SD of BoM. The limit of detection (LoD) was derived with the following formula: LoD = LoB + 1.645 SDp, where SDp is the SD of BNP values in patients with low BNP concentrations. Limits of quantification (LoQ) at 10% and 20% coefficient of variation (CV) were estimated by measuring three samples for BNPc and nine samples for BNPn, with BNP values ranging from 5 ng/L to 50 ng/L in different runs. To evaluate the within-run reproducibility of both methods, we used three samples with different mean BNP levels (54.9 ng/L, 119.3 ng/L, and 1113.1 ng/L for BNPn, and 41.7 ng/L, 403.4 ng/L, and 1478.8 ng/L for BNPc). We measured those samples 10 times each in the same run. We also evaluated between-run reproducibility. We used two samples with different mean BNP levels (113.4 ng/L and 1070.2 ng/L for BNPn, and 404.4 ng/L and 1490.9 ng/L for BNPc), and we measured BNP concentration once a day for two weeks.

Statistical analyses were performed using JMP version 14.0 (SAS Institute Inc., Cary, NC) and R software version 4.0.4 (R Foundation for Statistical Computing, Vienna).

## Results

### Patient characteristics

Clinical characteristics of study cohorts are summarized in Table 1.

**Table 1:** Patient characteristics (n = 1204).

Variables	
Age (year)	70 (IQR: 60 to 77)
Men/women	705/499
Body surface area (m^2^)	1.60 (1.47 to 1.74)
Body mass index (kg/m^2^)	22.4 (20.1 to 25.1)
SBP (mmHg)	130 (115 to 146)
DBP (mmHg)	75 (66 to 85)
Heart rate (bpm)	75 (65 to 85)
Hypertension (%)	614 (51%)
Atrial fibrillation (%)	154 (13%)
Dialysis	77 (6%)
Creatinine (mg/dL)	0.86 (0.68 to 1.14)
eGFR (mL/min/1.73m^2^)	61.2 (43.7 to 75.1)
Hb (g/dL)	12.5 ± 2.2
CRP (mg/dL)	0.15 (0.04 to 0.73)
Albumin (g/dL)	4.0 (3.5 to 4.3)
LVEF (%)	55 (50.5 to 57.5)

Continuous data are expressed as means ± standard deviations or medians and interquartile ranges (IQR). Categorical data are presented as absolute values and percentages.

CRP, C reactive protein; DBP, diastolic blood pressure; eGFR, estimated glomerular filtration rate; Hb, hemoglobin; IQR, interquartile range; LVEF, left ventricular ejection fraction.

### Analytical performance and reproducibility

LoB, LoD, and LoQ values of BNPc and BNPn are summarized in Table 2. We could not calculate LoQ values of BNPc because we could not estimate the interpolated reciprocal curve, perhaps due to the small sample size and the narrow range of BNP levels. Within-run reproducibility values of BNPc assay ranged from 2.0% for the sample with a medium BNP level to 2.5% for a sample with the lowest BNP level. Corresponding values of the BNPn assay ranged from 1.2% for the sample with the highest BNP level to 3.1% for the sample with the lowest BNP level. The between-run reproducibility of BNPn was 1.9% for the sample with lowest BNP level and 2.4% for the sample with highest BNP level. Corresponding values for BNPc were 2.8% and 2.9%.

**Table 2:** Summary of analytical performance values.

	LoB (ng/L)	LoD (ng/L)	LoQ	LoQ
			20%CV (ng/L)	10%CV (ng/L)
BNPc				
Present study	1.7	3.7	NA	NA
Manufacturer	NA	2.0	2.5	NA
BNPn				
Present study	0.0	2.2	3.8	10.9
Manufacturer	NA	7.6	NA	NA

BNPc, brain natriuretic peptide concentration by conventional method; BNPn, brain natriuretic peptide concentration by novel method; CV, coefficient of variation; LoB, limit of blank; LoD, limit of detection; LoQ, limit of quantification, NA; not available.

### Correlation between BNP levels by the conventional and novel methods

**Fig 1:**
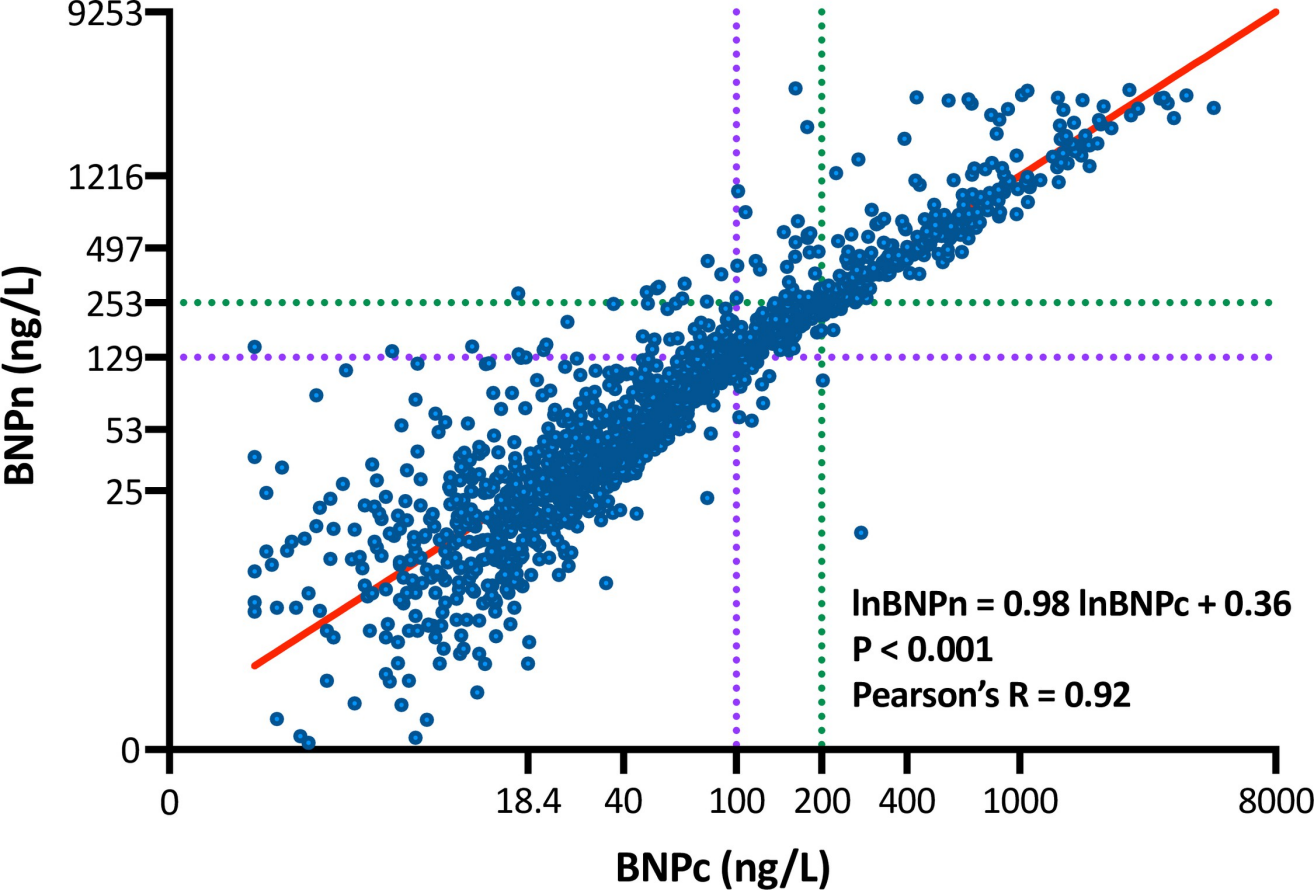
Correlation of brain natriuretic peptide (BNP) concentrations by conventional and novel methods. The X-axis denotes BNP concentration by the conventional method (BNPc), while the Y-axis denotes BNP concentration by the novel method (BNPn). The regression line is shown in red. Both axes are log-transformed. Tick marks on the Y axis corresponded to those on the X axis, according to the regression formula. For example, the green dotted line indicates that BNPn = 253ng/L corresponds to BNPc = 200ng/L, and the purple dotted line indicates that BNPn = 129ng/L corresponds to BNPc = 100ng/L.

Median values of BNPc and BNPn were 49 ng/L (IQR: 21 to 131 ng/L) and 71 ng/L (IQR: 26 to 193 mL), respectively. Fig 1 depicts the correlation between log-transformed BNPc (lnBNPc) and log-transformed BNPn (lnBNPn). Pearson’s coefficient of correlation between log-transformed BNP levels by both methods was 0.92 (p<0.001). A paired analysis showed that lnBNPn was significantly higher than lnBNPc (p<0.001). A linear regression analysis provided the following regression formula:

lnBNPn=0.98xlnBNPc+0.36.

### Concordance rate

Patients were divided into two groups based on a predefined BNPc cut-off value of 200 ng/L because most previous studies used this cut-off, which indicates a high likelihood of heart failure requiring treatment [9–11]. In contrast, a value of 250 ng/L was employed as the cut-off for BNPn, according to the regression formula described above (Fig 1). The concordance rate of group classification between BNPc and BNPn was 0.96, and Cohen’s kappa coefficient was 0.86 (p<0.001).

### Outcomes

**Fig 2:**
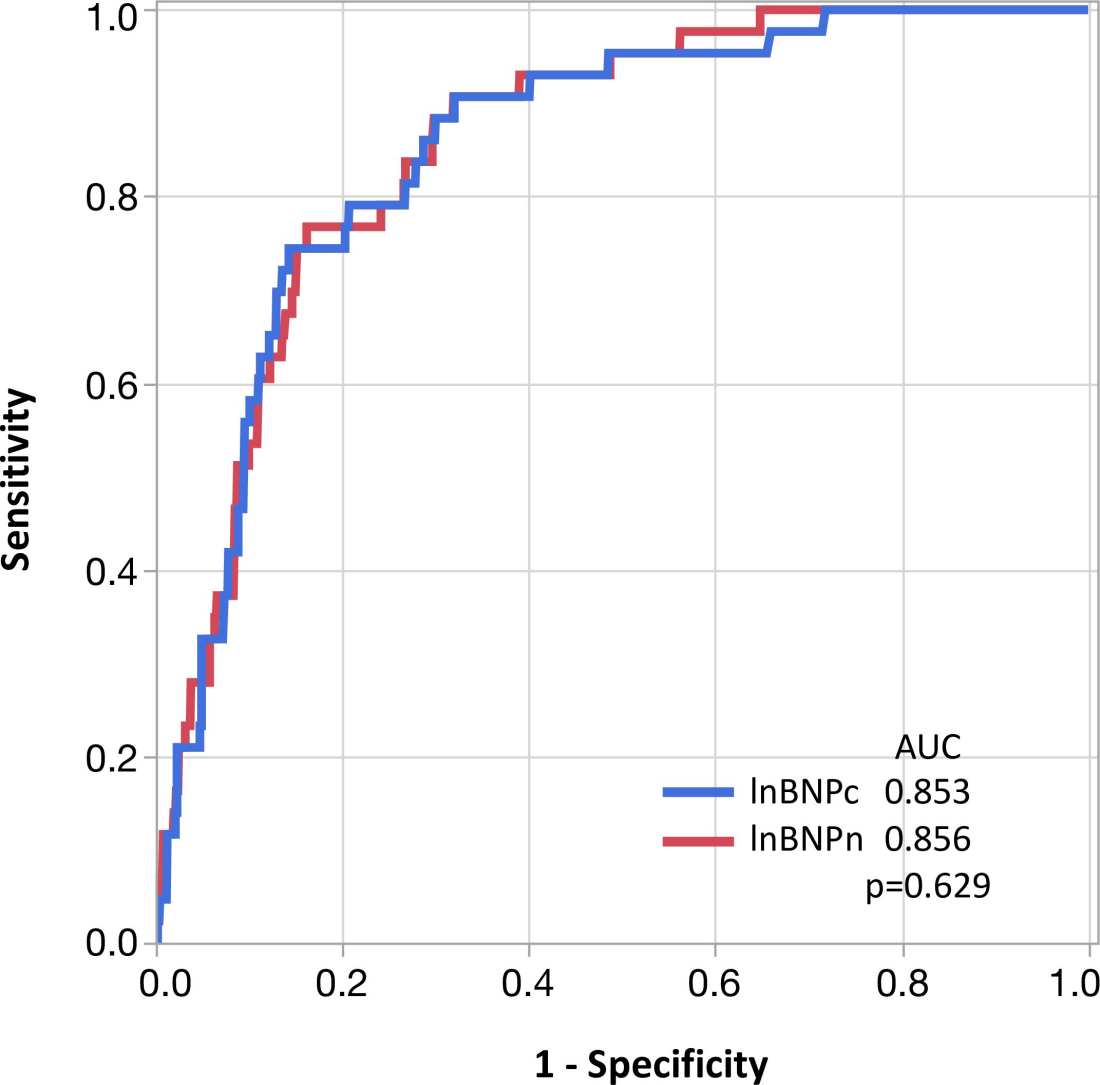
Receiver operation characteristic curves for prediction of cardiac events. Comparison of the area under the curve (AUC) of log-transformed brain natriuretic peptide by the conventional method (BNPc) and the novel method (BNPn) for cardiac events.

**Fig 3:**
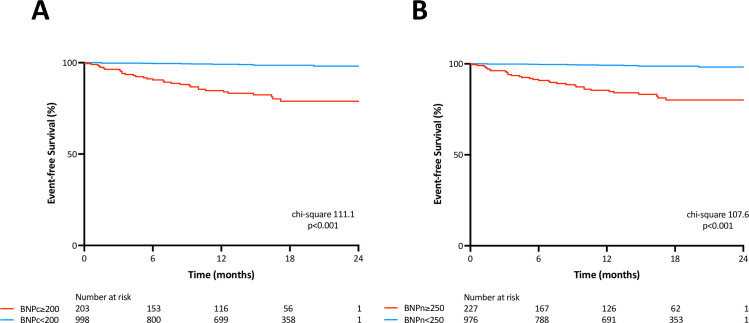
Kaplan-Meier survival analysis for cardiac events. A: Brain natriuretic peptide by the conventional method (BNPc), B: Brain natriuretic peptide by a novel method (BNPn). Patients were classified into two groups according to the cut-off values. Note that different cut-off values were employed, 200 ng/L for BNPc and 250 ng/L for BNPn.

During a median follow-up of 15.4 months (IQR: 7.9 to 19.4 months), 43 patients developed MACEs, including 15 cases of cardiac death, 25 cases of HF requiring hospitalization, 2 cases of non-fatal MI, and 3 cases of ventricular tachyarrhythmia. The sensitivity, specificity, and accuracy for a cut-off value of BNPc = 200ng/L for association of MACEs were 74%, 85%, and 85%, respectively. The corresponding values of BNPn = 250ng/L were 76%, 83%, and 83%, respectively. McNemar’s test revealed that the novel method has similar sensitivity, but lower specificity compared with the conventional method (Sensitivity: p = 0.317; Specificity; p = 0.003). Fig 2 depicts the comparison of ROC curves of lnBNPc and lnBNPn for prediction of MACEs. DeLong’s test revealed that AUCs of lnBNPc and lnBNPn were similar (p = 0.629). Fig 3A and 3B show the MACE-free survival rate between two groups stratified according to BNPc and BNPn cut-off values. The log-rank test revealed that there were significant survival differences between the two groups using both BNP criteria. Univariable Cox proportional Hazard analysis revealed that age, diastolic blood pressure, left ventricular ejection fraction, albumin, creatinine, C-reactive protein, hemoglobin, hypertension, atrial fibrillation, and lnBNPs were associated with MACEs (Table 3). Table 4A and 4B show the results of multivariable Cox proportional Hazard analyses. According to results from univariate analysis and clinical importance, we made three models for multivariate analysis. LnBNPn was significantly associated with MACEs even after adjusting other parameters (Table 4A), as was LnBNPc (Table 4B).

**Table 3:** Univariable Cox regression analyses of predictors of cardiac events.

	HR	95% CI	Z score	P value
Age (per 1 y.o increase)	1.041	1.014–1.070	2.935	0.003
Sex (Male)	1.776	0.926–3.406	1.730	0.084
BSA (per 1 m^2^ increase)	1.376	0.409–4.628	0.515	0.606
BMI (per 1 kg/m^2^ increase)	1.008	0.942–1.079	0.227	0.820
SBP (per 1 mmHg increase)	0.985	0.970–1.001	-1.879	0.060
DBP (per 1mmHg increase)	0.966	0.943–0.991	-2.714	0.007
Heat Rate (per 1 bpm increase)	1.004	0.983–1.024	0.341	0.733
Hypertension	2.846	1.435–5.646	2.993	0.003
Atrial fibrillation	2.554	1.312–4.974	2.758	0.006
Dialysis	2.261	0.954–5.357	1.853	0.063
Creatinine (per 1 mg/dL increase)	1.177	1.069–1.295	3.294	<0.001
eGFR (per 1 mL/min increase)	0.970	0.959–0.982	-5.132	<0.001
Hb (per 1 g/dL increase)	0.869	0.758–0.997	-2.005	0.045
CRP (per 1 mg/dL increase)	1.090	1.024–1.160	2.705	0.007
Albumin (per 1 g/dL increase)	0.425	0.286–0.632	-4.231	<0.001
LVEF (per 1% increase)	0.922	0.903–0.942	-7.541	<0.001
lnBNPc (per 2.718 ng/L increase)	2.165	1.808–2.591	8.412	<0.001
lnBNPn (per 2.718 ng/L increase)	2.400	1.946–2.960	8.188	<0.001

BMI, body mass index; lnBNPc, log-transformed brain natriuretic peptide concentration by conventional method; lnBNPn, log-transformed brain natriuretic peptide concentration by novel method; BSA, body surface area; CI, confidence interval; CRP, C reactive protein; DBP, diastolic blood pressure; eGFR, estimated glomerular filtration rate; HR, hazard ratio; Hb, hemoglobin; IQR, interquartile range; LVEF, left ventricular ejection fraction.

**Table 4:** **A**: Multivariable Cox regression analyses of predictors of cardiac events incorporating LnBNPn. **B**: Multivariable Cox regression analyses of predictors of cardiac events incorporating LnBNPc.

**A**						
	Comorbidity model		Hemodynamic model		Biomarker model	
	HR (95% CI)	P value	HR (95% CI)	P value	HR (95% CI)	P value
Age	1.017 (0.990–1.045)	0.209				
Hypertension	1.248 (0.607–2.566)	0.547	1.480 (0.732–2.994)	0.275		
Atrial fibrillation	1030 (0.520–2.040)	0.932	0.998 (0.497–2.006)	0.996		
LVEF			0.964 (0.940–0.989)	0.005		
Albumin					0.876 (0.491–1.563)	0.654
Creatinine					0.880 (0.742–1.043)	0.140
CRP					0.961 (0.880–1.050)	0.378
LnBNPn	2.347 (1.879–2.931)	<0.001	1.975 (1.527–2.554)	<0.001	2.565 (1.901–3.461)	<0.001
**B**						
	Comorbidity model		Hemodynamic model		Biomarker model	
	HR (95% CI)	P value	HR (95% CI)	P value	HR (95% CI)	P value
Age	1.019 (0.992–1.048)	0.172				
Hypertension	1.348 (0.659–2.758)	0.414	1.618 (0.803–3.260)	0.179		
Atrial fibrillation	1.088 (0.552–2.148)	0.807	1.081 (0.540–2.163)	0.827		
LVEF			0.960 (0.936–0.986)	0.002		
Albumin					0.766 (0.429–1.367)	0.367
Creatinine					0.911 (0.773–1.073)	0.263
CRP					0.997 (0.911–1.092)	0.954
LnBNPc	2.123 (1.750–2.576)	<0.001	1.770 (1.402–2.236)	<0.001	2.118 (1.662–2.699)	<0.001

lnBNPc, log-transformed brain natriuretic peptide concentration by conventional method; lnBNPn, log-transformed brain natriuretic peptide concentration by novel method; CI, confidence interval; CRP, C reactive protein; HR, hazard ratio; LVEF, left ventricular ejection fraction.

### Utility of assays in patients with lower BNP level

Patients were divided into two groups based on the predefined BNPc cut-off value of 100 ng/L because the Food and Drug Administration (FDA) of the United States recommends this cut-off value for early diagnosis of HF. A value of 130 ng/L was employed as the cut-off for BNPn, according to the regression formula described above (Fig 1). The concordance rate of group classification between BNPc and BNPn was 0.94, and Cohen’s kappa coefficient was 0.86 (p<0.001).

**Fig 4:**
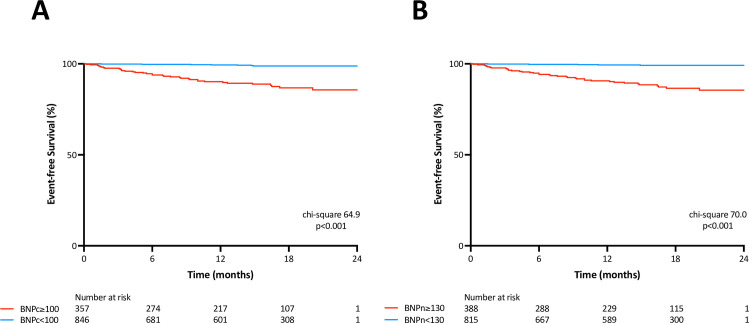
Kaplan-Meier survival analysis for cardiac events. A: Brain natriuretic peptide by the conventional method (BNPc), B: Brain natriuretic peptide by the novel method (BNPn). Patients were classified into two groups according to cut-off values. Note that different cut-off values were employed, 100 ng/L for BNPc and 130 ng/L for BNPn.

The sensitivity, specificity, and accuracy for a cut-off value of BNPc = 100ng/L for association of MACEs were 84%, 72%, and 73%, respectively. Corresponding values of BNPn = 130ng/L were 88%, 70%, and 70%, respectively. McNemar’s test revealed that the novel method has similar sensitivity, but lower specificity compared with the conventional method (Sensitivity: p = 0.157; Specificity; p<0.001). Fig 4A and 4B show the MACE-free survival rate between two groups stratified according to BNPc and BNPn cut-off values. The log-rank test revealed that there were significant survival differences between the two groups using both BNP criteria.

## Discussion

The major findings of this study are summarized as follows: 1) Log-transformed BNPc and BNPn were reasonably correlated; 2) However, BNPn levels were significantly higher than BNPc levels; 3) A similar association with future MACEs was observed by both methods.

Recent HF clinical practice guidelines give a Class 1 standing for natriuretic peptides for diagnosing and predicting the prognosis of HF [12, 13]. Especially, natriuretic peptides are useful to diagnose HF patients in the emergency room (ER). It is often challenging to differentiate HF patients from those who also complained in the ER of dyspnea due to other causes [1, 5, 14, 15]. A previous retrospective study reported that plasma BNP concentration was helpful in diagnosing HF in 269 patients who visited the ER due to dyspnea, and the authors revealed that the best cut-off value of BNP was 234 ng/L with a sensitivity of 87% and a specificity of 86% for HF diagnosis [16]. Despite this clinical usefulness of BNP testing, some hospitals in Japan cannot measure BNP concentration due to lack of an immunoassay analyzer.

N-terminal BNP (NT-proBNP) is also one of the natriuretic peptides for which clinical usefulness has been established [12, 13]. NT-proBNP is well correlated with BNP and is useful for diagnosing HF and predicting future cardiac events [17, 18]. NT-proBNP is usually measured using a general-purpose, automatic biochemical analyzer, which is used in most hospitals in Japan. As Clerico et al. previously reported, NT-proBNP measurement has some strengths compared to BNP measurement: 1) it is more stable in plasma and has a longer biological half-life, 2) there is a less systematic difference between immunoassay methods, 3) there is a more significant increase in circulating levels of the peptide from an early phase of heart failure, 4) the user does not need to consider usage of angiotensin receptor-neprilysin inhibitors, which could increase BNP levels [19, 20]. However, the NT-proBNP level is more readily affected by renal dysfunction and less sensitive to rapid hemodynamic changes [21]. Moreover, BNP testing is more used than NT-proBNP testing in Japan [22]. This is the main reason why we believe that the novel BNP assay using a general-purpose automatic biochemical analyzer has the potential to help many physicians in decision-making.

The analytical performance and reproducibility of BNPn were nearly equal to that of BNPc. BNPn was reasonably correlated with BNPc in regard to some outliers (Pearson’s r = 0.92). Collin-Chavagnac et al. previously reported a head-to-head comparison of 4 BNP assays that require an immunoassay analyzer [23]. According to their report, the Pearson’s correlation coefficient ranged from 0.929 to 0.994 among the four assays, and our result is comparable. However, we found that BNPn was significantly higher than BNPc. Although the exact reason for this phenomenon is speculative, one possible reason is a non-specific reaction due to the nature of the latex agglutination method. Collin-Chavagnac et al. also reported significant differences in slopes ranging from 0.80 to 1.84 among BNP concentrations by four BNP assays [23]. Similarly, Funatsuki et al. compared 12 assays which are available in the Japanese market incorporating Centaur XP and Nanopia BNP-A [24]. They used same clinical specimens for each reagent. The slopes of the regression formula to immunoradiometric method were from 0.88 to 1.88. Considering the relationship between BNPc and BNPn, we set specific cut-offs based on the linear regression formula. Then, the concordance rate and kappa coefficient of classification using specific cut-offs were excellent (0.96 and 0.86).

Survival analyses revealed that both BNPc and BNPn were significantly associated with future cardiac events. The BNPn cut-off value of 250 ng/L stratified the high-risk group of patients for adverse outcomes. Multivariate Cox proportional Hazard regression analyses revealed that both BNPc and BNPn were significantly associated with MACEs, even after adjusting cardiac and renal function, which indicates that the novel BNP kit using biochemical equipment has equivalent accuracy for predicting future outcomes compared with standard immunological BNP measurements.

We also analyzed the diagnostic and prognostic utility of lower BNPn levels (BNPc = 100 ng/L, BNPn = 130 ng/L) because the FDA recommends a cut-off of BNP = 100 ng/L for early detection of HF. The results were very similar to the higher cut-off values (BNPc = 200 ng/L, BNPn = 250 ng/L), and BNPn seems to be useful even in patients with lower BNP concentrations.

### Study limitations

There were several limitations to this study that should be addressed. First, this was a retrospective observational study from a single center. Second, our cohort includes all subjects who were clinically indicated for BNP testing. Thus, our subjects included asymptomatic patients. However, the absence of typical symptoms and physical signs in patients with heart failure is not rare, especially in elderly people [25]. Our study design, which included all patients for whom BNP measurements were ordered, could be the strength of this study in terms of generalizability. Third, we measured BNPn at a median of 251 days after BNPc measurement using a frozen residual sample. Because BNP is not stable in plasma even if the sample was frozen [19], a prospective study that allows measuring BNP by both methods at the same time is required to verify our results. Moreover, the difference in measurement date could be one of the reasons for the significant difference in BNP concentration between the two methods. Fourth, observed results may be applicable only for one of the BNP immunoassays. As previously reported, BNP concentrations using various immunoassays are not interchangeable [23]. Thus, the immunoassay that we used as a control in this study should be taken into consideration to interpret our results. Fifth, the more extensive use of BNP in Japan could limit the importance of this study, especially in areas where NT-proBNP is more commonly used. Sixth, the described LoB, LoD, LoQ, and reproducibility of both two methods were not completely based on international recommendations. Data were according to the pre-determined analyses we did when we started this study; however, we could not re-determine those parameters prospectively because our hospital had already replaced the immunoassay analyzer from Centaur XP with another machine. Further studies are required to assess the analytical performance of both assays based on a solid method. Finally, we could not determine the exact reason why BNPn levels were higher than corresponding BNPc levels. Further refinement of the biochemical assay for BNP may be required to resolve this issue.

## Conclusions

BNP levels were measured using a novel kit for general-purpose automatic biochemical analyzers were higher than BNP levels by the conventional method. However, BNP levels by the novel method provided equivalent prognostic information for future cardiac events compared with BNP levels using the conventional method. We believe that this method has the potential to expand clinical usage of BNP measurements, especially in hospitals that do not have an immunoassay analyzer.

## Supporting information

S1 File (XLSX)

## References

[1] Davis M, Espiner E, Richards G, Billings J, Town I, Neill A, et al. Plasma brain natriuretic peptide in assessment of acute dyspnoea. Lancet. 1994;343(8895):440–4. Epub 1994/02/19. doi: 10.1016/s0140-6736(94)92690-5 . 7905953

[2] Logeart D, Thabut G, Jourdain P, Chavelas C, Beyne P, Beauvais F, et al. Predischarge B-type natriuretic peptide assay for identifying patients at high risk of re-admission after decompensated heart failure. J Am Coll Cardiol. 2004;43(4):635–41. Epub 2004/02/21. doi: 10.1016/j.jacc.2003.09.044 . 14975475

[3] Suzuki S, Yoshimura M, Nakayama M, Mizuno Y, Harada E, Ito T, et al. Plasma level of B-type natriuretic peptide as a prognostic marker after acute myocardial infarction: a long-term follow-up analysis. Circulation. 2004;110(11):1387–91. Epub 2004/09/09. doi: 10.1161/01.CIR.0000141295.60857.30 . 15353502

[4] Weber M, Hamm C. Role of B-type natriuretic peptide (BNP) and NT-proBNP in clinical routine. Heart. 2006;92(6):843–9. Epub 2006/05/16. doi: 10.1136/hrt.2005.071233 ; PubMed Central PMCID: PMC1860679. 16698841 PMC1860679

[5] Dao Q, Krishnaswamy P, Kazanegra R, Harrison A, Amirnovin R, Lenert L, et al. Utility of B-type natriuretic peptide in the diagnosis of congestive heart failure in an urgent-care setting. J Am Coll Cardiol. 2001;37(2):379–85. Epub 2001/02/24. doi: 10.1016/s0735-1097(00)01156-6 . 11216950

[6] Maisel AS, Krishnaswamy P, Nowak RM, McCord J, Hollander JE, Duc P, et al. Rapid measurement of B-type natriuretic peptide in the emergency diagnosis of heart failure. N Engl J Med. 2002;347(3):161–7. Epub 2002/07/19. doi: 10.1056/NEJMoa020233 . 12124404

[7] Mueller T, Gegenhuber A, Poelz W, Haltmayer M. Head-to-head comparison of the diagnostic utility of BNP and NT-proBNP in symptomatic and asymptomatic structural heart disease. Clin Chim Acta. 2004;341(1–2):41–8. Epub 2004/02/18. doi: 10.1016/j.cccn.2003.10.027 . 14967157

[8] Musetti V, Masotti S, Prontera C, Storti S, Ndreu R, Zucchelli GC, et al. Evaluation of the analytical performance of a new ADVIA immunoassay using the Centaur XPT platform system for the measurement of cardiac troponin I. Clin Chem Lab Med. 2018;56(9):e229–e31. Epub 2018/04/22. doi: 10.1515/cclm-2018-0054 . 29679526

[9] Troughton RW, Frampton CM, Yandle TG, Espiner EA, Nicholls MG, Richards AM. Treatment of heart failure guided by plasma aminoterminal brain natriuretic peptide (N-BNP) concentrations. Lancet. 2000;355(9210):1126–30. Epub 2000/05/03. doi: 10.1016/s0140-6736(00)02060-2 . 10791374

[10] Packer M. Should B-type natriuretic peptide be measured routinely to guide the diagnosis and management of chronic heart failure? Circulation. 2003;108(24):2950–3. Epub 2003/12/17. doi: 10.1161/01.CIR.0000109205.35813.8E . 14676134

[11] Maisel A, Hollander JE, Guss D, McCullough P, Nowak R, Green G, et al. Primary results of the Rapid Emergency Department Heart Failure Outpatient Trial (REDHOT). A multicenter study of B-type natriuretic peptide levels, emergency department decision making, and outcomes in patients presenting with shortness of breath. J Am Coll Cardiol. 2004;44(6):1328–33. Epub 2004/09/15. doi: 10.1016/j.jacc.2004.06.015 . 15364340

[12] Yancy CW, Jessup M, Bozkurt B, Butler J, Casey DE Jr., Colvin MM, et al. 2017 ACC/AHA/HFSA Focused Update of the 2013 ACCF/AHA Guideline for the Management of Heart Failure: A Report of the American College of Cardiology/American Heart Association Task Force on Clinical Practice Guidelines and the Heart Failure Society of America. J Am Coll Cardiol. 2017;70(6):776–803. Epub 2017/05/04. doi: 10.1016/j.jacc.2017.04.025 . 28461007

[13] McDonagh TA, Metra M, Adamo M, Gardner RS, Baumbach A, Bohm M, et al. 2021 ESC Guidelines for the diagnosis and treatment of acute and chronic heart failure. Eur Heart J. 2021;42(36):3599–726. Epub 2021/08/28. doi: 10.1093/eurheartj/ehab368 . 34447992

[14] Stevenson LW, Perloff JK. The limited reliability of physical signs for estimating hemodynamics in chronic heart failure. JAMA. 1989;261(6):884–8. Epub 1989/02/10. 2913385

[15] Morrison LK, Harrison A, Krishnaswamy P, Kazanegra R, Clopton P, Maisel A. Utility of a rapid B-natriuretic peptide assay in differentiating congestive heart failure from lung disease in patients presenting with dyspnea. J Am Coll Cardiol. 2002;39(2):202–9. Epub 2002/01/15. doi: 10.1016/s0735-1097(01)01744-2 . 11788208

[16] Nakata K, Komukai K, Yoshii Y, Miyanaga S, Kubota T, Kosuga T, et al. The Optimal Cut-off Value of Plasma BNP to Differentiate Heart Failure in the Emergency Department in Japanese Patients with Dyspnea. Intern Med. 2015;54(23):2975–80. Epub 2015/12/04. doi: 10.2169/internalmedicine.54.4786 . 26631879

[17] Nabeshima Y, Sakanishi Y, Otani K, Higa Y, Honda M, Otsuji Y, et al. Estimation of B-type Natriuretic Peptide Values from N-Terminal proBNP Levels. J UOEH. 2020;42(1):1–12. Epub 2020/03/28. doi: 10.7888/juoeh.42.1 . 32213738

[18] Lainchbury JG, Campbell E, Frampton CM, Yandle TG, Nicholls MG, Richards AM. Brain natriuretic peptide and n-terminal brain natriuretic peptide in the diagnosis of heart failure in patients with acute shortness of breath. J Am Coll Cardiol. 2003;42(4):728–35. Epub 2003/08/23. doi: 10.1016/s0735-1097(03)00787-3 . 12932611

[19] Clerico A, Passino C, Franzini M, Emdin M. Cardiac biomarker testing in the clinical laboratory: where do we stand? General overview of the methodology with special emphasis on natriuretic peptides. Clin Chim Acta. 2015;443:17–24. Epub 2014/06/18. doi: 10.1016/j.cca.2014.06.003 . 24937843

[20] Clerico A, Zaninotto M, Passino C, Plebani M. New issues on measurement of B-type natriuretic peptides. Clin Chem Lab Med. 2017;56(1):32–9. Epub 2017/08/16. doi: 10.1515/cclm-2017-0433 . 28809748

[21] Srisawasdi P, Vanavanan S, Charoenpanichkit C, Kroll MH. The effect of renal dysfunction on BNP, NT-proBNP, and their ratio. Am J Clin Pathol. 2010;133(1):14–23. Epub 2009/12/22. doi: 10.1309/AJCP60HTPGIGFCNK . 20023254

[22] Fuji Keizai Co. L. 2020 Rinsyokensa Shijo No.1 Immunoassay Shijo (Clinical Examination Market 2020, No.1-Immunoassay Market): Fuji Keizai; 2020.

[23] Collin-Chavagnac D, Dehoux M, Schellenberg F, Cauliez B, Maupas-Schwalm F, Lefevre G, et al. Head-to-head comparison of 10 natriuretic peptide assays. Clin Chem Lab Med. 2015;53(11):1825–37. Epub 2015/05/23. doi: 10.1515/cclm-2014-0592 . 25996187

[24] Funatsuki K, MASUTA K, INOUE Y, NISHI H, IGARASHI M, HORII K, et al. Evaluation of Domestic BNP Measurement Reagents. Rinsho Byori. 2019;67(11):1103–8. PubMed PMID: 2020368803.

[25] Lien CT, Gillespie ND, Struthers AD, McMurdo ME. Heart failure in frail elderly patients: diagnostic difficulties, co-morbidities, polypharmacy and treatment dilemmas. Eur J Heart Fail. 2002;4(1):91–8. Epub 2002/01/29. doi: 10.1016/s1388-9842(01)00200-8 . 11812669

